# Online Measurement Method and System of Excitation Impedance of Current Transformers Based on Norton’s Theorem and Differential Method to Measure Difference of Two Currents

**DOI:** 10.3390/s24103115

**Published:** 2024-05-14

**Authors:** Mengying Gan, Hongsen You, Jiansheng Yuan

**Affiliations:** Department of Electrical Engineering, Tsinghua University, Beijing 100084, China; yhs20@mails.tsinghua.edu.cn

**Keywords:** current transformer, excitation impedance, Norton’s theorem, online measurement

## Abstract

An online measurement method is proposed in this paper, and a system is established for detecting the excitation impedance of current transformers (CTs) based on Norton’s theorem. The theorem is carried out by connecting a resistance and an inductance at the secondary side port of the CT to get the equations for calculating the impedance. The iterative method is used to solve the equations, and the solution is revised to consider the nonlinearity of the core. The main variable in the equations is the variation of the secondary current with the resistance or inductance. To obtain the secondary current variation accurately, which is less than 1‰ of the current, a differential method is proposed, which is based on charging two capacitors and measuring the difference of their voltages instead of measuring each current separately first and then obtaining the current variation by subtraction. This is equivalent to saving two currents first and then measuring the current difference. The differential method avoids the problem of error amplification in the process of measuring two currents separately first and then subtracting them to obtain the current variation and solves the problem that two currents do not appear simultaneously. The results verify the correctness and accuracy of the proposed method and system. The acquisition of the excitation impedance is the basis for obtaining the working characteristics of CT cores, including magnetic and loss characteristics, as well as the error of CTs.

## 1. Introduction

The working characteristics of current transformer (CT) cores are influenced by the exciting current. According to the CT equivalent circuit, the exciting current refers to the current that flows through the excitation impedance branch. The excitation reactance corresponds to the magnetic characteristics of the core, which mainly affects the CT ratio error; the excitation resistance corresponds to the loss characteristics of the core, which mainly affects the CT phase displacement. Therefore, measuring the excitation impedance serves as the foundation for determining the working characteristics of the core and detecting the CT errors.

Error detection plays a critical role in ensuring accurate measurement performance of CTs. The current transformer (CT) error measurement method recommended by the International Electrotechnical Commission (IEC) standard is the direct measurement method, also known as the comparison measurement method [1]. Its basic principle is to compare the measured CT with a standard CT with a higher accuracy class to determine the ratio error and phase displacement. Numerous studies have been devoted to improving the measurement range and accuracy of the comparison method [2,3,4,5].

It is impractical to apply the comparison method for on-site CT error detection, primarily because the large current source is difficult to build [6]. However, it is also laborious to remove the CTs and bring them back to the laboratory for calibration. To address this issue, the IEC standard offers an alternative method for on-site error measurement, which obtains the ratio error by offline measurement of the CT exciting current [1]. Both the comparison method and the alternative method necessitate service interruption for CT removal, calibration, and reinstallation, leading to high economic losses and waste of labor. Consequently, numerous CTs have remained uncalibrated for several years or even decades [7,8].

An effective online calibration method for CTs is necessary. It can not only overcome the aforementioned shortcomings of the offline calibration but also obtain the CT error data during real-time operation. Research in this area can be classified into two categories. One is based on the standard CT [9,10], which often involves manual operation of the clamp CT with low security [11,12]. The other is based on a phasor measurement unit (PMU) and complex algorithms [13,14]. These existing online calibration methods are difficult to be promoted and applied due to their complicated implementation and high cost.

This paper aims to propose an online measurement method and system for determining the excitation impedance of the CT based on Norton’s theorem. This method enables a novel approach for online detection of CT errors and core working characteristics [15]. The equations for solving the excitation impedance are established by simply connecting the resistance and inductance at the secondary winding port. By measuring only two voltage effective values at the secondary side, the excitation impedance can be obtained. Then the CT error can be calculated based on the excitation impedance. The proposed system is simple, effective and cost-effective.

## 2. Measuring Principle

**Figure 1:**
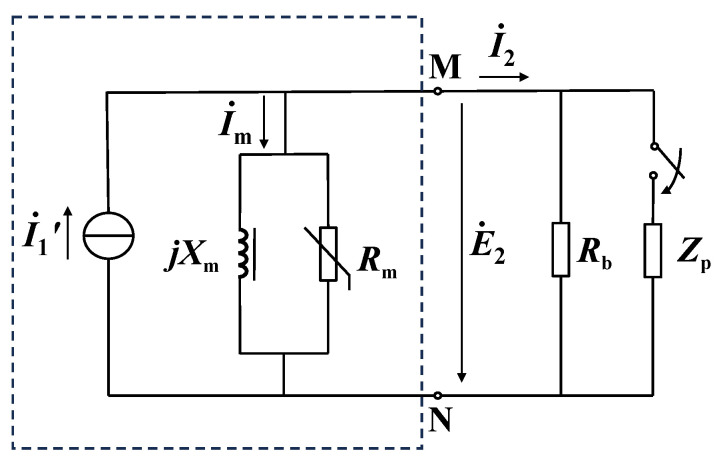
The equivalent circuit for measuring CT excitation impedance based on Norton’s theorem.

The dashed box in Figure 1 represents the CT equivalent circuit converted to the secondary side. In Figure 1, ${\dot{I}}_{1}^{\prime }$ is the converted primary current, ${\dot{I}}_{m}$ is the exciting current, ${\dot{I}}_{2}$ is the secondary current, *R*_b_ is the load resistance, *R*_m_(*E*_2_) is the nonlinear excitation resistance, *X*_m_(*E*_2_) is the nonlinear excitation reactance, and *E*_2_ is the secondary electromotive force (EMF), which is approximately equal to the voltage of the secondary port. Definitely, the flux of the core is a function of the currents ${\dot{I}}_{1}^{\prime }$ and ${\dot{I}}_{2}$, and ${\dot{I}}_{2}$ is a function of the load impedance *Z*_b_, so the excitation impedance is a function of *Z*_b_. In measurement, only the CT secondary voltage can be obtained, and the voltage drop of the winding resistance and leakage inductance needs to be added to obtain *E*_2_. The winding resistance needs to be measured in advance, while the leakage inductance is not easy to measure. The influence of leakage inductance is ignored in our measurement system.

As shown in Figure 1, the Norton equivalent impedance from the secondary side port MN is the excitation impedance *Z*_m_, and the equivalent current source is ${\dot{I}}_{1}^{\prime }$. By connecting two different impedances *Z*_p_ at the port and combining with the state of not connecting *Z*_p_, three effective value equations can be established. Then *R*_m_, *X*_m_, and $I_{1}^{\prime }$ can be obtained.

### 2.1. Selection Method for Properties and Values of Port-Connecting Impedances

Selecting the two port-connecting impedances as one resistance *R*_p_ and one reactance *X*_p_ is the best configuration so that the variation of the secondary current *I*_2_ can reflect the excitation impedance *Z*_m_ to the maximum extent. This is because the excitation resistance *R*_m_ can be reflected to the maximum extent when connecting *R*_p_ at the port, while the excitation reactance *X*_m_ can be reflected to the maximum extent when connecting *X*_p_ at the port. Then essentially, the equations based on Norton’s theorem are enabled to have a greater degree of linear independence, the amount of information contained is larger, and the accuracy of *Z*_m_ obtained by the measured secondary current variation ∆*I*_2_ is higher.

**Figure 2:**
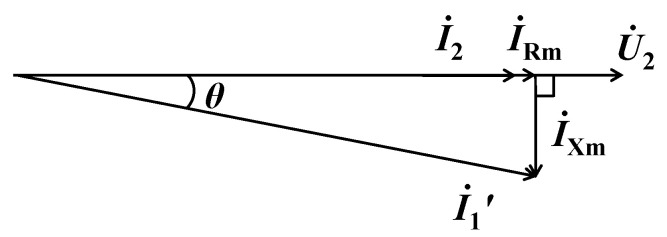
Phasor diagram of CT.

The phenomenon that ∆*I*_2_ causes by the port-connecting resistance *R*_p_ mainly reflects the excitation resistance *R*_m_, and ∆*I*_2_ caused by the port-connecting reactance *X*_p_ mainly reflects *X*_m_ and can be explained by the phase relationship of each current. The phasor diagram when not connecting *R*_p_ or *X*_p_ is shown in Figure 2. Since the load is a resistor *R*_b_ (IEC standard stipulates that the load is a resistor of 0.2 Ω when detecting CT errors [1]), *I*_2_ is in phase with the current *I*_Rm_ flowing through *R*_m_ and has a 90-degree angle with the current *I*_Xm_ flowing through *X*_m_. Under the excitation of the constant current source $I_{1}^{\prime }$, if *R*_p_ is connected at the port, *I*_2_ will increase slightly, while *θ* in Figure 2 is basically unchanged. The increase in *I*_2_ is basically equal to the decrease in *I*_Rm_, and *I*_Xm_ is basically unchanged. Therefore, the port-connecting resistance *R*_p_ mainly reflects the excitation resistance *R*_m_. Calculation shows that the variation of *I*_Rm_ is two orders of magnitude larger than that of *I*_Xm_. Correspondingly, when *X*_p_ is connected at the port, *I*_2_ will increase, and *θ* will also increase significantly. Thus, the variation of *I*_Xm_ is greater than that of *I*_Rm_. Due to the constraint of the load resistance *R*_b_, the variation of *I*_Xm_ is not two orders of magnitude larger than that of *I*_Rm_ when connecting *X*_p_, but it can generally be several times larger.

For the numerical selection of *R*_p_, from Norton’s theorem, the smaller *R*_p_ the better, because a smaller *R*_p_ enables ∆*I*_2_ to be larger. Namely, the amount of information provided is larger, and it is easy to accurately measure. However, the magnetic flux in the core will decrease after connecting *R*_p_, and then both *R*_m_ and *X*_m_ will decrease due to the nonlinearity of the core so *Z*_m_ cannot be solved accurately by using a set of Norton’s theorem equations. In this sense, the greater *R*_p_ the better, because its impact on the core will be smaller. Therefore, the selection of *R*_p_ should be a compromise between providing enough information to facilitate measurement and making the core flux change small. Although we propose the iterative revision method for the nonlinearity of the iron core (see Section 2.3), the calculation accuracy is still affected by the degree of change of the ferromagnetic flux. Combined with the accuracy of the measurement and the accuracy of the calculation method considering the nonlinearity of the iron core, we suggest that the *R*_p_ including the lead resistance should be about 0.5 times the load resistance *R*_b_. For the value of *X*_p_, it is obviously not that the smaller *X*_p_ the better, due to the parallel constraint of *R*_b._ A smaller *X*_p_ will weaken the effect of port-connecting inductance. For a comprehensive consideration, we suggest that *X*_p_ is about 1.5*R*_b_.

### 2.2. Forms of Three Equations

When not connecting *Z*_p_, the secondary current is denoted as ${\dot{I}}_{2O}$, and the EMF is denoted as ${\dot{E}}_{2O}$. Then the equation is

(1) $${\dot{I}}_{1}^{\prime }=\frac{{\dot{E}}_{2O}}{R_{m}(E_{2O})}+\frac{{\dot{E}}_{2O}}{jX_{m}(E_{2O})}+{\dot{I}}_{2O}.$$

Taking the modulus of both sides of (1), the following equation is obtained:

(2) $$I_{1}^{\prime }={\left(\frac{E_{2O}^{2}}{R_{m}{\left(E_{2O}\right)}^{2}}+\frac{2I_{2O}E_{2O}}{R_{m}(E_{2O})}+I_{2O}^{2}+\frac{E_{2O}^{2}}{X_{m}{\left(E_{2O}\right)}^{2}}\right)}^{\frac{1}{2}}.$$

When connecting *R*_p_, the secondary current is denoted as ${\dot{I}}_{2R}$, and the EMF is denoted as ${\dot{E}}_{2R}$. Then the equation is

(3) $${\dot{I}}_{1}^{\prime }=\frac{{\dot{E}}_{2R}}{R_{m}(E_{2R})}+\frac{{\dot{E}}_{2R}}{jX_{m}(E_{2R})}+{\dot{I}}_{2R}.$$

Taking the modulus of both sides of (3), the following equation is obtained:

(4) $$I_{1}^{\prime }={\left(\frac{E_{2R}^{2}}{R_{m}{\left(E_{2R}\right)}^{2}}+\frac{2I_{2R}E_{2R}}{R_{m}(E_{2R})}+I_{2R}^{2}+\frac{E_{2R}^{2}}{X_{m}{\left(E_{2R}\right)}^{2}}\right)}^{\frac{1}{2}}.$$

When connecting *X*_p_, the secondary total impedance is denoted as *Z*_2X_, where *Z*_2X_ = *R*_b_ // *jX*_p_ = *R*_2X_ + *jX*_2X_. The secondary current is denoted as ${\dot{I}}_{2X}$, and the EMF is denoted as ${\dot{E}}_{2X}$. Then the equation is

(5) $${\dot{I}}_{1}^{\prime }=\frac{{\dot{E}}_{2X}}{R_{m}(E_{2X})}+\frac{{\dot{E}}_{2X}}{jX_{m}(E_{2X})}+{\dot{I}}_{2X}.$$

Taking the modulus of both sides of (5), the following equation can be sorted out:

(6) $$I_{1}^{\prime }={\left(\frac{E_{2X}^{2}}{R_{m}{\left(E_{2X}\right)}^{2}}+\frac{2I_{2X}^{2}R_{2X}}{R_{m}(E_{2X})}+I_{2X}^{2}+\frac{E_{2X}^{2}}{X_{m}{\left(E_{2X}\right)}^{2}}+\frac{2I_{2X}^{2}X_{2X}}{X_{m}(E_{2X})}\right)}^{\frac{1}{2}}.$$

Equations (2), (4) and (6) are the three effective value equations. However, the quantities being sought are actually not three including *R*_m_, *X*_m_, and $I_{1}^{\prime }$, but seven, because the secondary electromotive forces are different in the three cases.

### 2.3. Solution Method of Excitation Impedance

We propose a two-step method for calculating *R*_m_ and *X*_m_ (i.e., *R*_m_(*E*_2O_) and *X*_m_(*E*_2O_)). The first step is to consider that the three sets of *R*_m_ and *X*_m_ are the same, namely ignoring nonlinearity, and to solve. The second step is to use the results of the first step as the initial values, combined with the magnetic characteristics *B*-*H* and loss characteristics *P*-*B* of the core material obtained by the online equivalent measurement of our measurement system, and perform iterative revision to achieve nonlinear processing.

#### 2.3.1. Solution of Considering the Three Sets of R_m_ and X_m_ as the Same

Assuming that the excitation impedance when connecting *R*_p_ or *X*_p_ is the same as that when not connecting *Z*_p_, that is, *R*_m_(*E*_2O_) and *X*_m_(*E*_2O_), then *R*_m_ and *X*_m_ can be calculated by Equations (2), (4) and (6) based on the measured values. By combining (2) and (4) first, an equation for calculating the excitation resistance *R*_m_ and the excitation reactance *X*_m_ can be derived:

(7) $$A_{R}R_{m}^{2}+(2I_{2R}E_{2R}-2I_{2O}E_{2O})R_{m}+E_{2R}^{2}-E_{2O}^{2}=0,$$

where:

(8) $$A_{R}=I_{2R}^{2}-I_{2O}^{2}+\frac{E_{2R}^{2}-E_{2O}^{2}}{X_{m}^{2}}.$$

All the variables in (7) and (8) except for *R*_m_ and *X*_m_ are measurable. Equation (7) appears to be straightforward to solve as it is a quadratic equation of *R*_m_. However, Equation (8) contains *X*_m_, necessitating the establishment of an additional equation to calculate *R*_m_ and *X*_m_.

Then, by combining (2) and (6), another equation for calculating *R*_m_ and *X*_m_ is derived:

(9) $$A_{X}X_{m}^{2}-2I_{2X}^{2}X_{2X}X_{m}+E_{2O}^{2}-E_{2X}^{2}=0,$$

where:

(10) $$A_{X}=I_{2O}^{2}-I_{2X}^{2}+\frac{2I_{2O}E_{2O}-2I_{2X}^{2}R_{2X}}{R_{m}}+\frac{E_{2O}^{2}-E_{2X}^{2}}{R_{m}^{2}}.$$

Hence, *R*_m_ and *X*_m_ can be calculated by combining (7) and (9). However, owing to the variation characteristics of the excitation impedance, these equations need to be solved using an iterative method. The iterative method requires selecting initial values, which can be determined as follows:

(11) $$R_{m}^{\left(0\right)}=\frac{\left|E_{2R}-E_{2O}\right|}{\left|I_{2R}-I_{2O}\right|},$$

(12) $$X_{m}^{\left(0\right)}=\frac{\left|E_{2X}-E_{2O}\right|}{\left|I_{2X}-I_{2O}\right|}.$$

Equation (11) provides the solution for *R*_m_ in (7) when assuming that *X*_m_ is not present. As the change in circuit state caused by connecting a resistance primarily reflects *R*_m_, *R*_m_ calculated by omitting *X*_m_ is relatively close to its actual value. Similarly, (12) yields the solution for *X*_m_ in (9) when assuming that *R*_m_ is absent.

The iterative solution process is as follows: the *X*_m_^(0)^ calculated by (12) is substituted into (7)–(8) as a known quantity to calculate the excitation resistance value *R*_m_^(1)^; the *R*_m_^(0)^ calculated by (11) is substituted into (9)–(10) as a known quantity to calculate the excitation reactance value *X*_m_^(1)^. The solution of the equations can be obtained by this iteration.

#### 2.3.2. Iterative Revision Method Considering the Core Nonlinearity

The relationship between the excitation impedance and the electromotive force is established first. According to the relationship between the loss of ferromagnetic materials and the magnetic density [16], and according to the function form of the magnetization curve [17], the function of *R*_m_ and *X*_m_ changing with the electromotive force *E*_2_ can be expressed as

(13) $$R_{m}(E_{2})={\left(a_{r}E_{2}^{b_{r}}+c_{r}\right)}^{-1},$$

(14) $$X_{m}(E_{2})={\left(a_{x}E_{2}+b_{x}\right)}^{\frac{1}{2}}+c_{x}.$$

Theoretically, the six constants in (13) and (14) can be obtained by measurements for the core material. However, the CT will work under different measured currents, which is equivalent to the measurement of the core. Therefore, we propose a method to determine the constants in the above two equations according to the states of CTs under different currents: form six equations from (13) and (14) using the measured *E*_2O_ and the calculated *R*_m_ and *X*_m_ under three different measured currents (the current difference cannot be too small) and solve six unknown constants. Certainly, the *R*_m_(*E*_2O_) and *X*_m_(*E*_2O_) at this time are not accurate, and the obtained constants are also not accurate, which requires the iteration described below to be revised.

After obtaining the function of *R*_m_ and *X*_m_ changing with *E*_2_, revise the *R*_m_(*E*_2O_) and *X*_m_(*E*_2O_) obtained in Section 2.3.1. The revision method is that the excitation resistance and reactance after considering the nonlinearity are expressed as

(15) $$R_{\mathrm{mR}}(E_{2R})=k_{R}R_{m}(E_{2O}),$$

(16) $$X_{\mathrm{mX}}(E_{2X})=k_{X}X_{m}(E_{2O}),$$

where *k*_R_ is the ratio of the two resistances obtained by using *E*_2O_ and *E*_2R_ (i.e., EMF when connecting *R*_p_) based on (13), similar to *k*_X_.

Substitute (15) and (16) into (4) and (6), and then start solving again from Section 2.3.1, including revising the six constants based on (13) and (14) and recalculating (15) and (16), to iteratively deal with the nonlinearity of the iron core. Table 1 presents an example to show the effect of the iterative revision method.

## 3. Measurement System of Excitation Impedance

### 3.1. System Configuration

**Figure 3:**
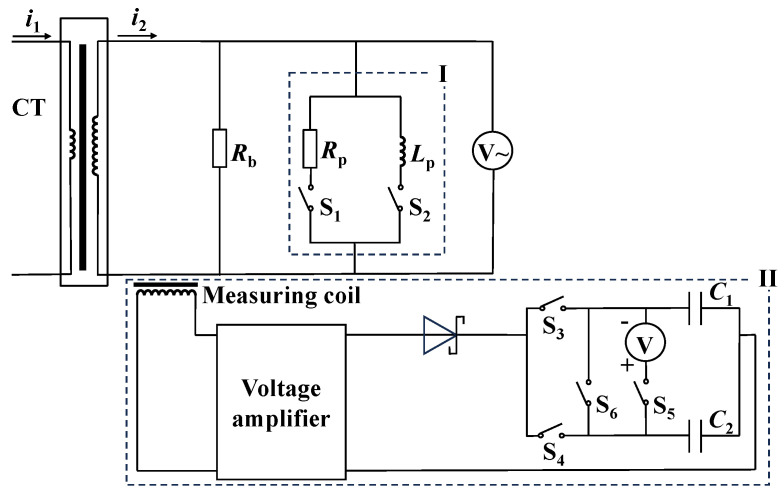
Configuration diagram of the measurement system.

The measurement system consists of two main modules, as illustrated in Figure 3. Module I is the port-connecting impedance switching module, and module II is the differential measurement module. The two port-connecting impedances are a resistor *R*_p_ and an inductor *L*_p_, and the reason for this setting has been previously discussed. The rated load resistance *R*_b_ of the CT is typically 0.2 Ω, and we have selected *R*_p_ as 0.1 Ω (including the converted resistance of the secondary winding and the connecting line) and *X*_p_ (equal to 2∗*π*∗50∗*L*_p_) as 0.3 Ω. This selection aims to maximize the secondary current variation when connecting *R*_p_ or *X*_p_ while accurately reflecting *R*_m_ or *X*_m_. If *R*_b_ is doubled or halved, the selected *R*_p_ and *X*_p_ should also be correspondingly increased or decreased. The subsequent discussion primarily focuses on the differential measurement module.

### 3.2. Necessity of Using Differential Method

Limited by *R*_b_, the total secondary impedance after connecting *R*_p_ or *L*_p_ can only decrease from *R*_b_, regardless of the selected values of *R*_p_ or *L*_p_. Furthermore, the excitation impedance is much greater than the total secondary impedance, leading to minimal variation in the secondary current *I*_2_ when connecting different impedances at the port, generally less than 1‰ of the secondary current.

For two quantities with a relative change of less than 1‰, if they are measured separately and are then subtracted to obtain the variation, the measurement error will be seriously enlarged. When two large numbers are subtracted to yield a small number, the error of the large numbers is enlarged, leading to a substantial error in the result.

For instance, if the actual value of the current before connecting the port-connecting impedance is *I*_2_ = 0.998 A, and the actual value after connecting the port-connecting impedance is *I*_2p_ = 0.999 A, then the variation between the two is Δ*I*_2_ = 0.999 − 0.998 = 0.001 A. Assume a measurement error of −0.1% for *I*_2_, namely its measured value is *I*_2d_ = 0.997 A, and if the measurement error of *I*_2p_ is zero, then the difference is Δ*I*_2d_ = 0.999 − 0.997 = 0.002 A. Consequently, the error of Δ*I*_2d_ relative to its actual value Δ*I*_2_ = 0.001 A reaches 100%.

As demonstrated above, it is difficult to obtain relatively accurate current variation and excitation impedance by separately measuring the secondary currents and then subtracting them. To address the issue of enlarging the error caused by subtracting two large numbers, we propose a differential method to directly measure the current variation Δ*I*_2_.

### 3.3. Principle and Implementation of Differential Method

The differential method is generally used to measure the difference between two simultaneous quantities. It is difficult to apply it to the measurement of secondary current variation due to the fact that the unchanged and changed secondary currents do not exist simultaneously but exist at different times.

To address this issue, we take the scheme of saving two currents first and then measuring the current difference. Obviously, it is difficult to directly save the current, so we use the method of converting the current to DC voltage and saving the voltage on the capacitors. Specifically, capacitors *C*_1_ and *C*_2_ are charged with the voltages corresponding to the unchanged and changed secondary currents respectively, and subsequently, the voltage difference between the two capacitors is measured by a voltmeter. The configuration of the two capacitors is depicted in the lower right corner of Figure 3. Once the capacitor is charged, the capacitor port is disconnected. The capacitor voltage can be maintained for a relatively long time and remains basically unchanged for a fraction of a second before the voltage difference is measured.

For measuring current transformers, the change rate of the primary current with time is generally not too large because power loads generally do not change suddenly, except at the electrical switching time, and this effect can be eliminated by data processing. Therefore, as long as the time of charging the two capacitors and measuring the voltage difference is as short as possible, $I_{1}^{\prime }$ can be considered basically unchanged in this process, and the secondary current variation and its corresponding voltage difference are merely caused by switching the port-connecting impedance.

The processes of charging and discharging the two capacitors shown in Figure 3 are as follows: Close S_3_ to charge *C*_1_ while keeping the other switches open. Next, open S_3_ and close S_4_ to charge *C*_2_. Subsequently, open S_4_ and close S_5_ to connect the DC millivolt meter to measure the voltage difference and record the voltage at the moment of connecting the voltmeter. Due to the voltmeter’s equivalent large resistance, the capacitor starts to discharge gradually after connecting the voltmeter, and the discharge time constant is the product of the capacitance and the internal resistance of the voltmeter, so the voltage at the moment of connection needs to be recorded. It can also be inferred that the capacitance selected should not be too small, otherwise, the time constant is too small. Then the discharge process is too fast to record the correct voltage. Moreover, a capacitor with excessively small capacitance is susceptible to external interference. After experimental verification, we select the Polypropylene Film Capacitor (CBB capacitor) with a capacitance of 10 μF. After recording the voltage, close S_6_ to neutralize the charges on the two capacitors. Finally, open S_6_. So far, the measurement of a capacitor charge and discharge cycle has been completed.

To enhance measurement accuracy, multiple measurement cycles should be promptly conducted, and then the data should simply be processed to determine the voltage difference. We take the average value of the voltage difference data measured in a single cycle as the result of a single measurement first. Then we take the average value of the voltage difference measured for 20 consecutive cycles and again take the average value of the 10 voltage differences closest to this average value as the final voltage difference result. The specific settings of the switch action time and other components of the differential module are as outlined below.

### 3.4. Differential Measurement Module Configuration

The main components of the differential measurement module include the current conversion coil, coil voltage amplifier, rectifier circuit, two capacitors, and switches, as depicted in Figure 3.

The secondary circuit wire of the measured CT passes through the current conversion coil, which converts the secondary current into coil port voltage. The coil port is connected to the voltage amplifier to amplify the voltage. The amplifier is connected to the rectifier circuit, and its output voltage typically exceeds 10 V at the CT-rated current. The rectifier circuit is linked with switches, capacitors, and the voltmeter.

The voltage across the secondary total impedance cannot directly reflect the secondary current because the secondary total impedance changes with the port-connecting impedance. The ratio of the port voltage of the current conversion coil to the secondary current can be called the volt–ampere ratio. To enhance the signal-to-noise ratio, the coil volt–ampere ratio should be relatively large, ideally set to 0.1. As the volt–ampere ratio of an air-core coil is approximately 10^−5^, a core-equipped coil is necessary. The turn number of the coil itself should be large, and the turn number of the CT secondary wire around the coil can also be increased appropriately. However, caution must be exercised to prevent core saturation, as the excitation current applied to the core constitutes the entire secondary current. We choose a core coil with a single-turn open-circuit voltage of 0.1 mV under the excitation magnetomotive force of 1 ampere-turn and set its turn number to 1000 to achieve a volt–ampere ratio of 0.1.

Since the rated secondary current of CT is 1 A or 5 A, the coil output voltage is less than 1 V, potentially insufficient to turn the rectifier diode conductive. We choose an OP37 operational amplifier to amplify the coil output voltage. Its input offset voltage is only at the microvolt level, the maximum input voltage is 2 V_pp_, the maximum magnification is 100, and the maximum output voltage is 13 V.

The amplified voltage can be rectified to charge the capacitor. Upon connection to the charging source, the capacitor swiftly reaches peak voltage. The rectifier diode is no longer conductive until disconnection because the capacitor is not connected to any load impedance and has no discharge path. Therefore, a rectifier circuit composed of a single rectifier diode can meet the requirements. We select the 1N5822 Schottky diode, which has a maximum forward voltage drop of 0.525 V.

It should be emphasized that the influence of these components on the overall accuracy of the system is difficult to obtain accurately by calculation. Therefore, the calibration method commonly used in measurements is used to calibrate the system so there is no need to explicitly establish the transfer function of each segment or precisely define the relationship between the input and output. The relationship between the input and output of the entire system can be obtained through calibration.

### 3.5. Implementation of Measurement System Based on PLC

**Figure 4:**
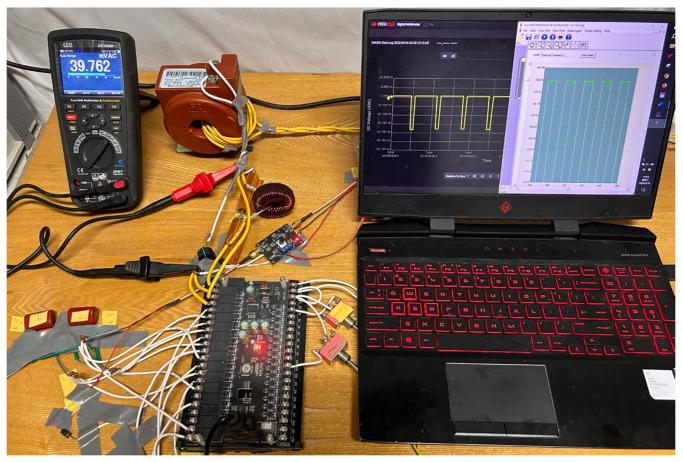
The implementation of the measurement system.

The switch action time must account for the time constant of the capacitor circuit to ensure adequate time for capacitor charging and discharging. Nonetheless, it is essential to limit the charging duration of the capacitors to prevent potential fluctuations in the primary current, which could compromise measurement accuracy. The programmable logic controller (PLC) is employed to realize switch control and system automatic measurement. The model number of the PLC used is EnYu-FX2N 32MR, which is manufactured by Shenzhen EnYu Technology Co., Ltd. The switches in Figure 3 are designated to distinct control ports of the PLC; that is, the switches S_1_~S_6_ in Figure 3 adopt the relay switches integrated into the PLC. The implementation of the measurement system is shown in Figure 4.

All switches are opened initially. In a single measurement of switching the port-connecting resistance (or inductance), the switch action sequence and delay are as follows: Close S_3_, delay 100 ms, and then open S_3_. Close S_1_ (or S_2_) and delay 80 ms. Close S_4_, delay 100 ms, and then open S_4_. Close S_5_, delay 300 ms, and then open S_5_. Open S_1_ (or S_2_). Close S_6_, delay 100 ms, and then open S_6_. Moreover, to ensure the normal execution of the program, a 20 ms delay is inserted between adjacent program steps because the relay switch requires a certain amount of action time.

The measurement system can be used as an affiliated module of an online CT to monitor its working status. However, the measurement only needs to be carried out once every few days or longer, and the measurement of each time only takes a few seconds. When not measuring, the system is electrically disconnected from the CT. It is obvious that the variation in the excitation impedance and CT error is a slow process, and the variation is generally very small over several months unless a sudden external force causes the CT to damage it.

It should be pointed out that remanence may occur in the CT core during its operation, which may be caused by the direct current component of the primary current, external magnetic field, or large current. Thus, demagnetization is necessary sometimes. Demagnetization can be achieved by injecting demagnetization current into the CT secondary loop circuit. The recommended maximum value of the demagnetization current is 200 mA. The accuracy of the CT with the largest error is generally 2%, and with the rated secondary current of 5 A, the exciting current is estimated to be 5 × 2% = 0.1. Thus, 200 mA is twice the exciting current, which is sufficiently large. Almost all the demagnetization current generates magnetic flux in the core because the impedance of the primary side is relatively large, and the primary side can be approximately considered as an open circuit seen from the secondary side. The frequency of the demagnetization current might be about 75 Hz to avoid overlapping with the working exciting current. To inject the demagnetization current into the CT secondary loop, the secondary current measurement coil of the CT measurement system can be used as a coupling coil to couple the current to the secondary circuit as the demagnetization current. When demagnetizing, the coil is disconnected from the current measurement circuit and connected to the demagnetizing power supply using automatic switches. The coil used in the system is a 1000-turn core coil so that as long as a 0.2 mA current is introduced to the coil, a current of about 200 mA can be coupled in the CT secondary circuit.

### 3.6. Characteristics of Differential Measurement Results

**Figure 5:**
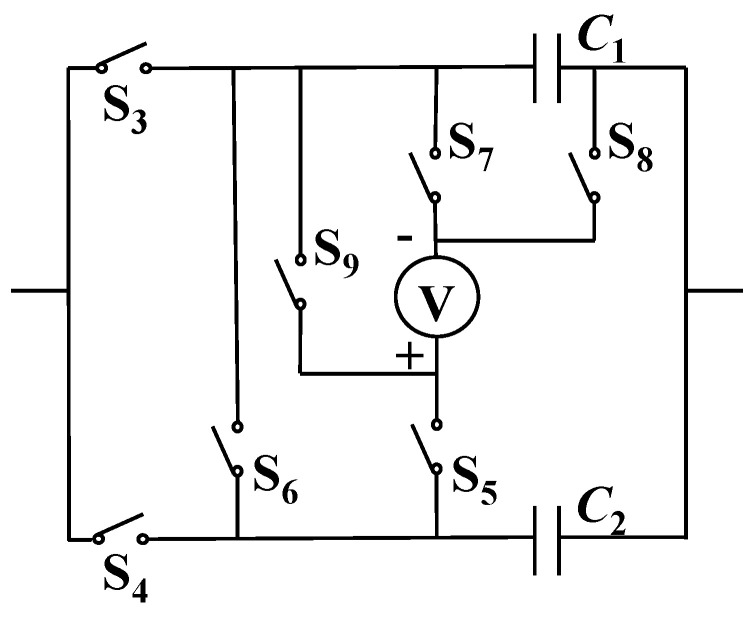
Switch connection to orderly measure the voltages on the two capacitors.

To observe the effect of the differential measurement, the charging voltage on each single capacitor could be first measured in turn. In addition to the previously mentioned switches S_1_~S_6_, switches S_7_~S_9_ are added, and all switches are opened initially, as shown in Figure 5. The switch action sequence corresponding to this measurement is as follows: Close S_3_ and then open S_3_. Close S_1_. Close S_4_ and then open S_4_. Close S_5_ and S_8_ and then open S_5_ and S_8_. Close S_9_ and S_8_ and then open S_9_ and S_8_. Open S_1_. Close S_6_ and then open S_6_.

**Figure 6:**
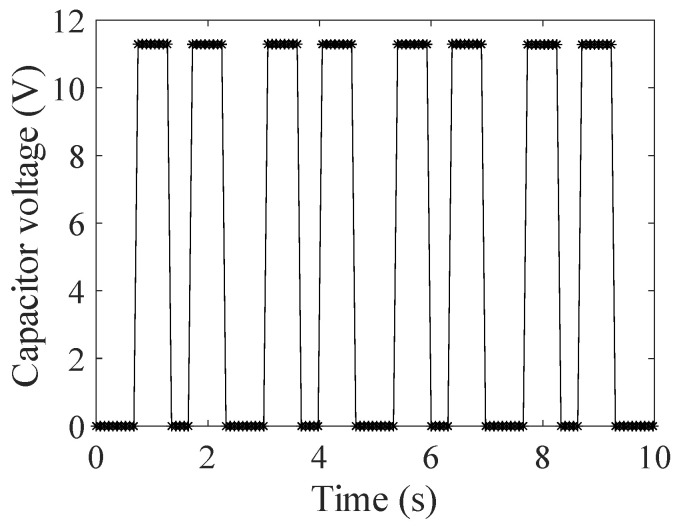
Measurement results of the voltages of the two capacitors.

**Figure 7:**
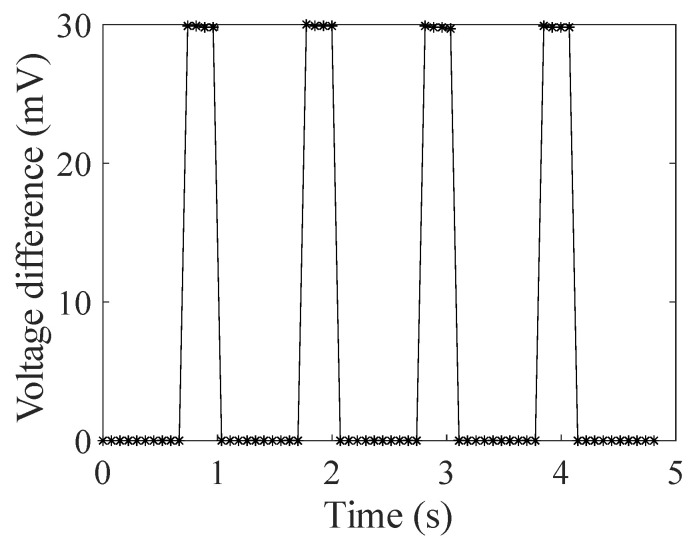
Measurement results of the voltage difference of the two capacitors.

Figure 6 shows the measurement results of the voltages of the two capacitors. In this figure, each cycle corresponds to the cycle of switching the port-connecting resistance and charging and discharging the capacitors, and the two pulses included correspond to the voltages on the capacitors *C*_2_ and *C*_1_, respectively. As can be seen from this figure, the voltage of a single capacitor is roughly 11 V. However, the naked eye cannot discern subtle differences between the two voltages of the two capacitors (the differences of the two pulses in each cycle). Similarly, the measurement results of the voltage difference of the two capacitors are shown in Figure 7. It can be easily seen that the voltage difference is roughly 30 mV, which is merely 0.3% of the voltage of a single capacitor. Therefore, the measurement system can effectively realize the differential measurement of minor variations of the secondary current.

## 4. System Calibration and Measurement Results

### 4.1. System Calibration

In the established system, the voltage difference Δ*U* of the two capacitors is measured, but the calculation of the excitation impedance requires the secondary current variation Δ*I*_2_. Thus, it is necessary to derive the relationship between Δ*I*_2_ and Δ*U*, which is represented by the proportional coefficient *k* = Δ*I*_2_/Δ*U*. Although this relationship is determined by the transformation ratio of the current conversion coil, the forward voltage drop of the rectifier diode and the magnification, it is difficult to calculate a relatively accurate *k* through separate measurement of these parameters. Therefore, the calibration method commonly employed in measurement is used to determine *k* by measurement. Since every component in the differential module is designed to function within the linear region, only one *k* value needs to be determined by calibration.

Theoretically, the Δ*I*_2_ should be directly fabricated, and then the Δ*U* could be measured. However, fabricating and accurately measuring Δ*I*_2_ is almost impossible because Δ*I*_2_ can only be obtained by measuring two currents separately and then subtracting them. The error amplification problem of the subtraction of two large numbers will occur in this process. Therefore, we use an indirect calibration method based on the impedance measured by the LCR instrument as a benchmark or an accurate value, that is, using the excitation impedance measured by the LCR instrument at the CT port as a benchmark. The CT is also measured with our measurement system, and the excitation impedance obtained by the measurement system is adjusted repeatedly. The calibration is completed when the excitation impedance obtained by the measurement system is the same as that of the LCR instrument, and the required *k* value is obtained.

Both the port-connecting resistor and inductor are utilizable for calibrating the system indirectly. We calibrate by using the port-connecting inductor. The expression of Δ*I*_2_ in terms of the electromotive force, excitation impedance, and secondary current is subsequently derived. By combining (2) and (6), but meanwhile accounting for the change of excitation impedance before and after connecting the port-connecting inductor, the expression of the secondary current variation Δ*I*_2_ = *I*_2X_–*I*_2O_ can be derived. The excitation impedance when not connecting the port-connecting impedance is denoted as *R*_mO_ and *X*_mO_, whereas the excitation impedance when connecting port-connecting inductor is represented as *R*_mX_ and *X*_mX_. These impedance values are accessible through an LCR impedance measuring instrument. Consequently, the expression of Δ*I*_2_ can be determined as follows:

(17) $$\Delta I_{2}=\frac{S_{R}+S_{X}}{\frac{E_{2O}}{R_{b}}+\frac{E_{2X}}{Z_{2X}}},$$

where:

(18) $$S_{R}=\frac{E_{2X}^{2}}{R_{\mathrm{mX}}^{2}}-\frac{E_{2O}^{2}}{R_{\mathrm{mO}}^{2}}-\frac{2E_{2O}^{2}}{R_{\mathrm{mO}}R_{b}}+\frac{2R_{2X}E_{2X}^{2}}{R_{\mathrm{mX}}Z_{2X}^{2}},$$

(19) $$S_{X}=\frac{E_{2X}^{2}}{X_{\mathrm{mX}}^{2}}-\frac{E_{2O}^{2}}{X_{\mathrm{mO}}^{2}}+\frac{2X_{2X}E_{2X}^{2}}{X_{\mathrm{mX}}Z_{2X}^{2}}.$$

In the formulas, *E*_2O_ and *E*_2X_ represent the secondary electromotive forces before and after connecting the port-connecting inductor, respectively.

**Figure 8:**
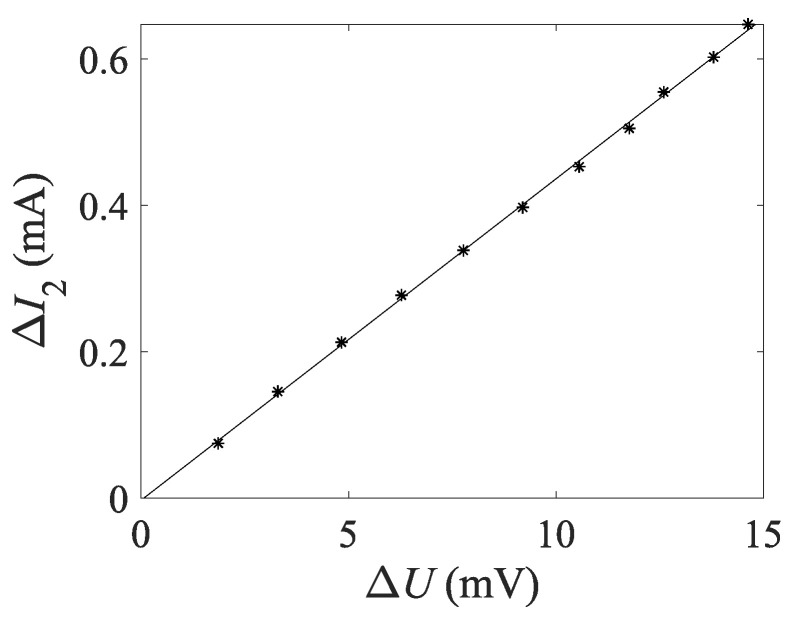
System calibration results.

Figure 8 illustrates the correlation between Δ*U* and Δ*I*_2_ at varying currents. A strong linear relationship is demonstrated between the two parameters. The coordinate ratio of any arbitrary point can be denoted as *k*. Furthermore, the mean value of each point can serve as the *k* value of the system. Here *k* is determined to be 0.044.

### 4.2. Excitation Impedance Measurement Results

The impedances of various CTs are measured by the system, and the results are compared with those obtained by the LCR impedance measuring instrument to verify the correctness and accuracy of the measurement system.

The process of the measurement is as follows: pass the primary wire through the CT; pass the secondary wire through the current conversion coil of the measurement system and connect it to the load resistance; connect the port-connecting impedance port of the measurement system in parallel with the load resistance and connect the digital voltmeter to the CT port. Adjust the current of the primary current source in turn and the computer collects and records the data of the measurement system. Calculate the excitation impedance based on these data. To compare with the results of the LCR instrument, the exciting current under different primary currents is calculated first, and the port impedance of the CT under different currents is then measured by using the current excitation gear of the LCR instrument, which is exactly the excitation impedance under different primary currents. Demagnetization should be carried out before measuring impedance.

In the actual measurement, the resistance of the winding and the lead cable should be taken into account to calculate the secondary total impedance. We calculate the lead resistance by collecting voltage and current from the CT port, but the winding resistance needs to be measured offline. If the lead between the CT and the load is long, the influence of the inductance of the lead circuit should also be considered, and the voltage and current of the CT port and their phase difference need to be measured. Our measurement system does not consider the lead inductance. We suggest that if the lead is long, the two wires should be wound (using the stranded wire) to weaken the influence of the inductance of the lead circuit.

**Figure 9:**
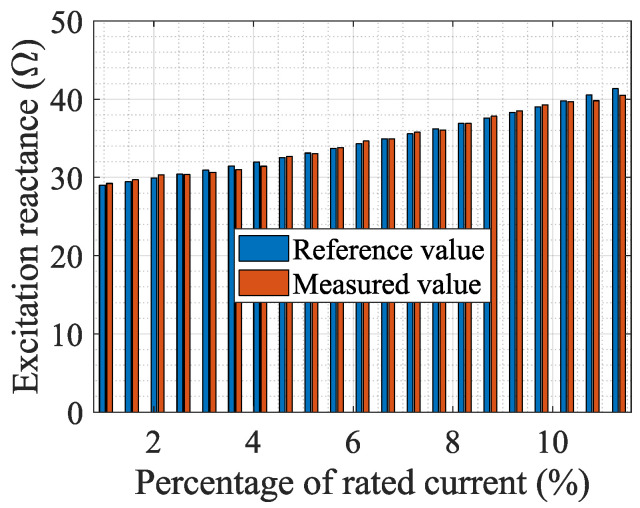
Measured and reference values of the excitation reactance.

**Figure 10:**
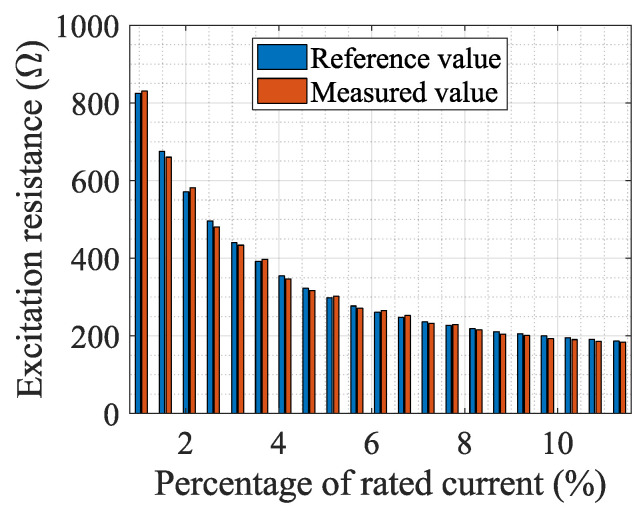
Measured and reference values of excitation resistance.

**Figure 11:**
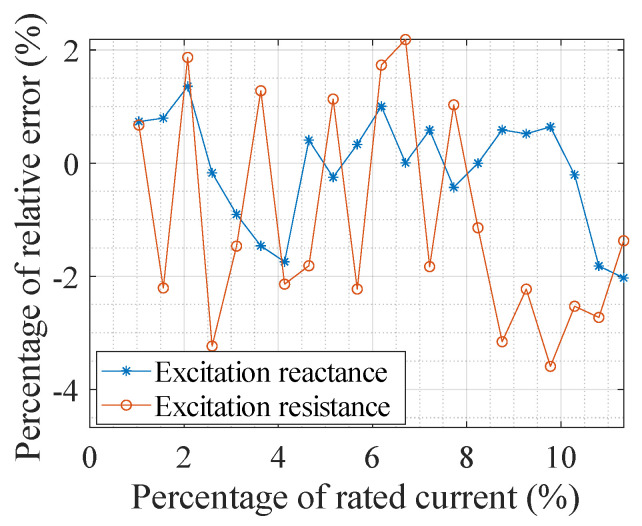
Relative error of the measured value to the reference value of excitation impedance.

To show the change of the CT port voltage when the port-connecting impedance is connected, Table 2 presents the measurement results of the port voltage (i.e., the EMF) of a 100/5 A CT under different currents, and the EMF is proportional to the magnetic flux in the iron core. In this table, *E*_2O_, *E*_2X_, and *E*_2R_ are, respectively, the electromotive forces when not connecting any impedance, connecting a reactance, and connecting a resistance at the CT port. Figure 9 and Figure 10 show the comparison of the measurement results of the excitation reactance and resistance obtained from the system with those obtained from the impedance measuring instrument. Additionally, Figure 11 illustrates the variance between the results obtained from the measurement system and the impedance measuring instrument. The measured CT is a 0.5 S class CT with a transformation ratio of 100/5 A. The resistance of the CT secondary winding is *R*_2r_ = 0.053 Ω, and the set load resistance is *R*_b_ = 0.2042 Ω.

## 5. Conclusions

In acquiring the CT excitation impedance by connecting two impedances at the secondary port, the selection of a reactor and a resistor as the impedances is the best choice. The former can reflect the excitation reactance to the maximum extent, while the latter reflects the excitation resistance. Since there is a load resistance of about 0.2 Ω at the port, an optimal port-connecting reactance of approximately 0.3 Ω is advised. A smaller port-connecting resistance enhances the effectiveness of reflecting the excitation resistance but meanwhile leads to a greater alteration in the core working state so that a port-connecting resistance of 0.1 Ω is deemed suitable. Since the excitation impedance is much larger than the load resistance, the secondary current variation caused by connecting the port-connecting impedances is minimal, less than 1‰. Meanwhile, the current variation is a key variable in the calculation of the excitation impedance. If the two secondary currents are measured separately and then subtracted to obtain the variation, the measurement error will be seriously enlarged. The differential method employing two capacitors proposed in this paper improves the measurement accuracy of the current variation effectively. The differences between the excitation impedance measurement results of the system developed in this paper and those obtained by the LCR impedance measuring instrument are less than 2% for the excitation reactance and less than 4% for the resistance.

## Figures and Tables

**Table 1:** An example to present the effect of the iterative revision method.

Round of the Revision	Calculated *R*_m_ (Ω)	Relative Error of *R*_m_ (%)	Calculated *X*_m_ (Ω)	Relative Error of *X*_m_ (%)
0	145.4875277	−23.7579	38.47930921	−5.089954389
1	184.9786893	−3.0627	39.89635446	−1.594781727
2	186.3426017	−2.3479	40.01671832	−1.297901683
3	186.8357202	−2.0895	40.03084616	−1.263055059

**Table 2:** Measurement results of the EMF under different currents.

*I*_2_ (mA)	*E*_2O_ (mV)	*E*_2X_/*E*_2O_	*E*_2R_/*E*_2O_
100.0104	25.7226	0.8351	0.3763
200.6962	51.6190	0.8358	0.3813
400.7554	103.0743	0.8366	0.3894
600.8822	154.5470	0.8375	0.4101
800.4978	205.8881	0.8386	0.4465

## Data Availability

The data presented in this study are available on request from the corresponding authors.
